# Evaluation of the Photodynamic Therapy with Curcumin on *L. braziliensis* and *L. major* Amastigotes

**DOI:** 10.3390/antibiotics10060634

**Published:** 2021-05-25

**Authors:** André Henrique Correia Pereira, Luciana Maria Cortez Marcolino, Juliana Guerra Pinto, Juliana Ferreira-Strixino

**Affiliations:** Photobios–Photobiology Applied to Health, University of Vale do Paraíba, Urbanova, São José dos Campos 2911, SP, Brazil; andre_gcp@hotmail.com (A.H.C.P.); lumaria.cortez@hotmail.com (L.M.C.M.); jgbiomd@hotmail.com (J.G.P.)

**Keywords:** photodynamic therapy, curcumin, amastigotes, macrophages, cutaneous leishmaniasis

## Abstract

Cutaneous leishmaniasis (CL) is a neglected disease prevalent in tropical countries with the ability to cause skin lesions. Photodynamic therapy (PDT) represents a specific and topical option for the treatment of these lesions. This study evaluated the response of macrophages infected with *L. braziliensis* and *L. major* to PDT with curcumin. Curcumin concentrations were evaluated in serial dilutions from 500.0 to 7.8 µg/mL using LED (λ = 450 ± 5 nm), with a light dose of 10 J/cm^2^. The Trypan blue viability test, ultrastructural analysis by scanning electron microscopy (SEM), mitochondrial polarity by Rhodamine 123 (Rho 123), curcumin internalization by confocal microscopy, and counting of the recovered parasites after the PDT treatment were performed. The lowest concentrations of curcumin (15.6 and 7.8 µg/mL) presented photodynamic inactivation. Cell destruction and internalization of curcumin in both macrophages and intracellular parasites were observed in microscopy techniques. In addition, an increase in mitochondrial membrane polarity and a decrease in the number of parasites recovered was observed in the PDT groups. This study indicates that PDT with curcumin has the potential to inactivate infected macrophages and might act as a basis for future in vivo studies using the parameters herein discussed.

## 1. Introduction

Cutaneous leishmaniasis (CL) is a neglected disease caused by a protozoan of the genus *Leishmania* spp., typical of tropical regions and commonly present in underdeveloped countries. WHO places CL as one of the 10 most important infectious diseases worldwide, being present in more than 85 countries, and worsening the situation of people who, due to the development of the disease, may have to leave their professional occupations due to the physical and psychosocial disorders they face [1,2,3].

The treatment of CL is done through the administration of pentavalent antimonials such as Pentosan^®^ and Glucantime^®^, or even with the use of Amphotericin B in more severe cases. However, these drugs present several side effects such as nausea, vomiting, and cardiac disorders, among others, besides presenting restrictions on their use by patients with heart problems or pregnant women [4,5].

Photodynamic therapy (PDT) is an alternative to the conventional treatment for CL, standing out for being non-invasive and presenting very few application restrictions for the patients. PDT is based on the inactivation of infected cells through the production of reactive oxygen species (ROS) from the photochemical process that results from the interaction between a photosensitizer (PS) and light at a wavelength suitable to it releasing oxygen [6,7].

CL is potentially more harmful to the most impoverished populations in developing countries, since its transmission usually affects individuals with poor access to health conditions, basic sanitation, and low purchasing power. Therefore, PDT may represent an important alternative since it presents minimum side effects and can be easily implemented. Clinical applications of PDT in CL treatment using PS, such as methyl-ALA, is widely reported, indicating the potential of this therapy [8,9,10]. However, it is important to consider that if the treatment cost is low, the access to the therapy for the poorest population will be greater, especially in underdeveloped countries such as Brazil, Colombia, Afghanistan, Peru, and Bolivia [1,11].

A feature that could make the application of PDT a less expensive method is the use of a low-cost and easily obtained PS, such as curcumin. This orange pigment compound is derived from *Curcuma longa* and is currently used as an oral supplement in the management of various dermatological conditions [12]. The spectrum of light absorption is between 430 and 500 nm, allowing PDT with curcumin to be applied superficially to skin lesions caused by *Leishmania* [13,14]. However, studies evaluating the use of this PS are still scarce, especially those involving the co-culture of macrophages, a condition that most closely resembles the clinical development of CL.

Considering the scarcity of studies evaluating the effects of curcumin as PS in the treatment of CL, this work aimed to evaluate the in vitro response of PDT with curcumin in the amastigotes of *L. braziliensis* and *L. major*, analyzing the inactivation of the infected macrophages, the interaction of the cells with curcumin, the cell morphology after the therapy, the mitochondrial alterations, and the retrieval of *Leishmania* after PDT.

## 2. Results

### 2.1. Viability Assessed by the Trypan Blue Exclusion Test

**Figure 1:**
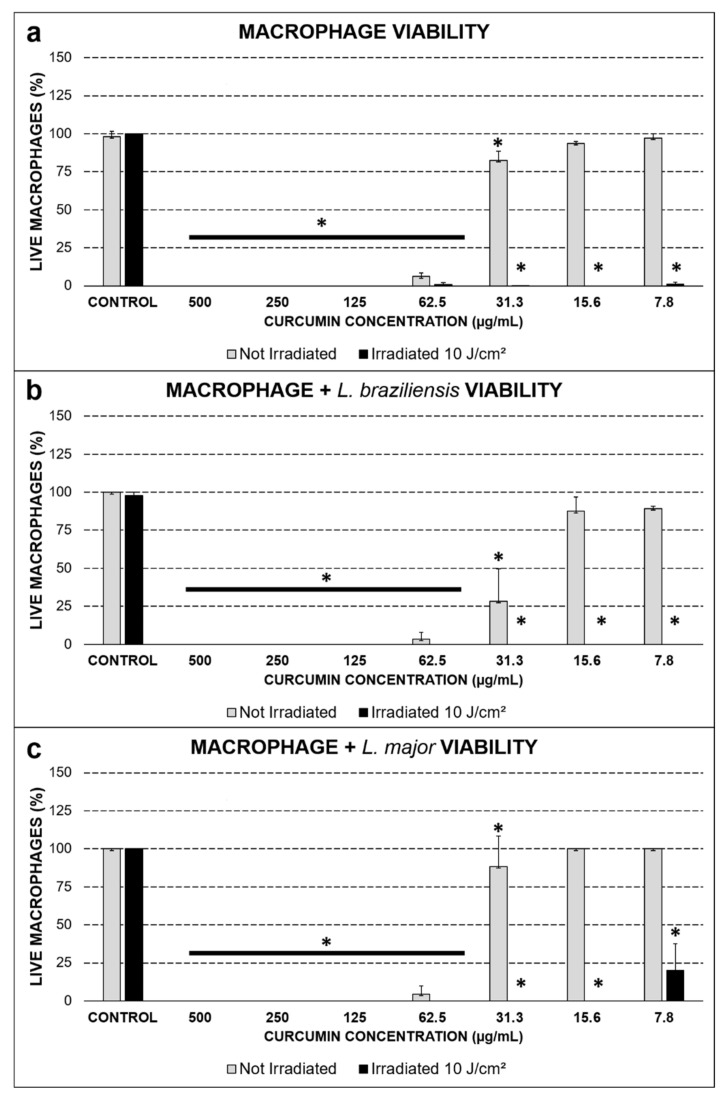
Viability test by the Trypan blue dye exclusion test, (**a**) uninfected macrophages, (**b**) macrophages infected with *L. braziliensis*, and (**c**) macrophages infected with *L. major*. Values are expressed as mean ± SD. The * symbol represents a statistical difference (*p* ≤ 0.01) between the control group and the other groups indicated with the same symbol. The horizontal lines represent the comparison of all of the groups below them.

In the viability test, the cytotoxic effect of curcumin in the dark was assessed at 500.0, 250.0, 125.0, and 62.5 µg/mL. This behavior was repeated when the uninfected macrophages were evaluated, as well as those infected with the two species of *Leishmania*, indicating that these concentrations were not interesting for in vitro application, since only curcumin in the dark resulted in cell death. However, at low PS concentrations, the viability of the groups kept in the dark was higher than 80% (*p* ≤ 0.01) at 15.6 and 7.8 µg/mL, and when submitted to PDT, the groups maintained viability below 5%, even reaching the complete inactivation of the macrophages infected with *L. braziliensis* and *L. major*. At the lowest concentration evaluated (7.8 µg/mL), the macrophages infected with *L. major* were slightly more resistant to PDT under these parameters (Figure 1).

### 2.2. Morphological Evaluation by Scanning Electron Microscopy

**Figure 2:**
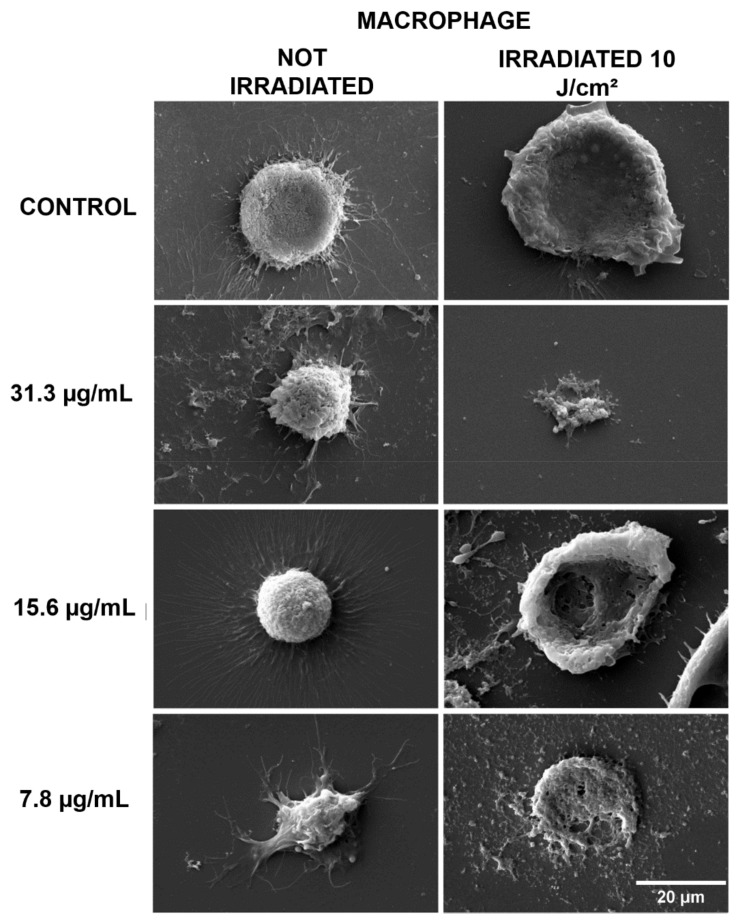
SEM of uninfected macrophages, with non-irradiated and irradiated groups. Control: cells that were not incubated with curcumin. The 31.3, 15.6, and 7.8 µg/mL groups were incubated with curcumin at these concentrations. Magnification of 2000×.

Considering the results obtained in the viability test, concentrations of 31.3, 15.6, and 7.8 µg/mL were selected to carry out the evaluation of cell morphology. In the groups kept in the dark, the characteristic morphology was observed, with no visible damage detected. In the groups that received PDT, intense cell destruction was observed, indicating that the changes occurred due to the action of this substance (Figure 2).

**Figure 3:**
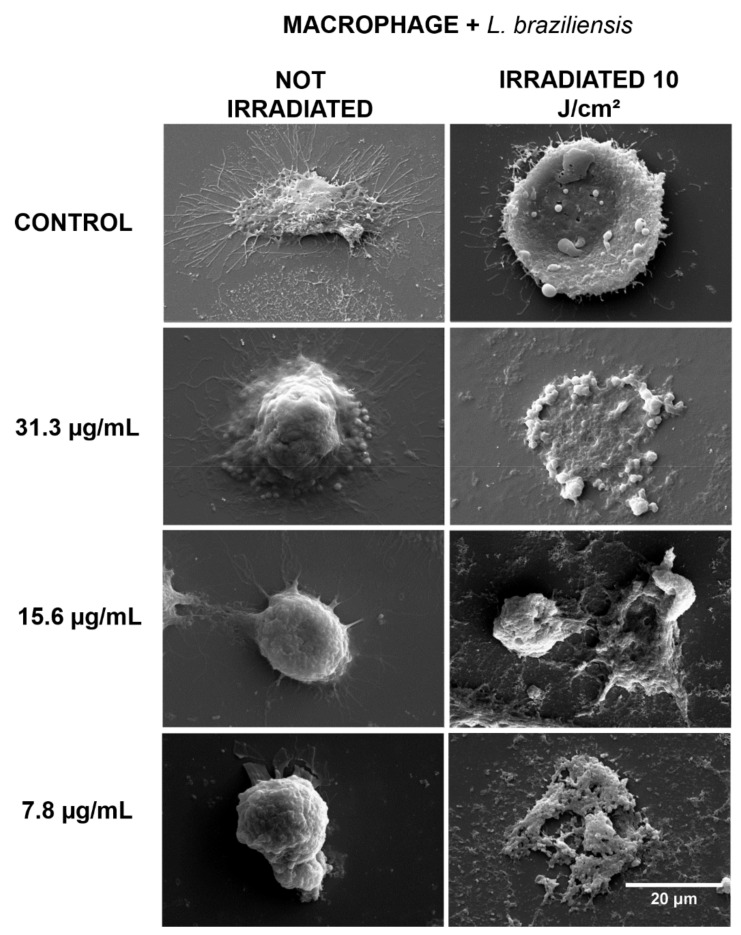
SEM for macrophages infected with *L. braziliensis*, with the non-irradiated and irradiated groups. Control: cells that were not incubated with curcumin. The 31.3, 15.6, and 7.8 µg/mL groups were incubated with curcumin at these concentrations. Magnification of 2000×.

**Figure 4:**
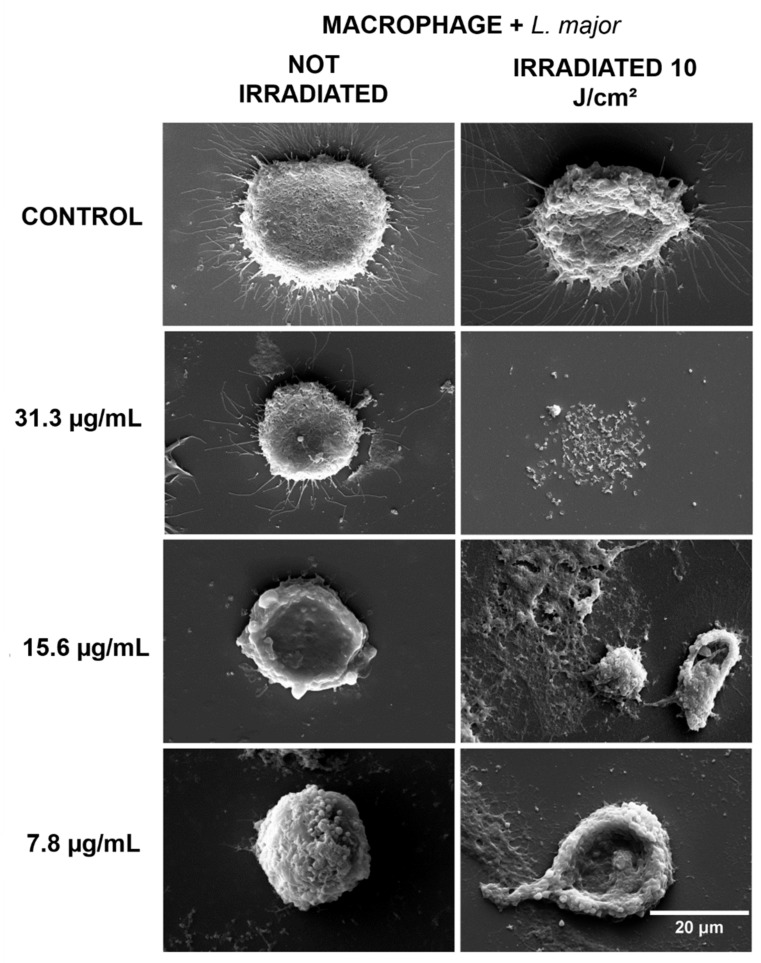
SEM for macrophages infected with *L. major*, with the non-irradiated and irradiated groups. Control: cells that were not incubated with curcumin. The 31.3, 15.6, and 7.8 µg/mL groups were incubated with curcumin at these concentrations. Magnification of 2000×.

Regarding the macrophages infected with *L. braziliensis* (Figure 3) and *L. major* (Figure 4) that were kept in the dark, a higher number of cytoplasmic prolongations was observed in the cells from the non-irradiated groups, in a process expected for phagocytic cells, such as macrophages, in the presence of foreign organisms like *Leishmania*. In the groups that received PDT, intense cellular destruction of infected macrophages was observed, confirming the inactivation promoted by PDT, as shown in the results of cell viability.

### 2.3. Internalization of Curcumin in Isolated and Leishmania-Infected Macrophages

**Figure 5:**
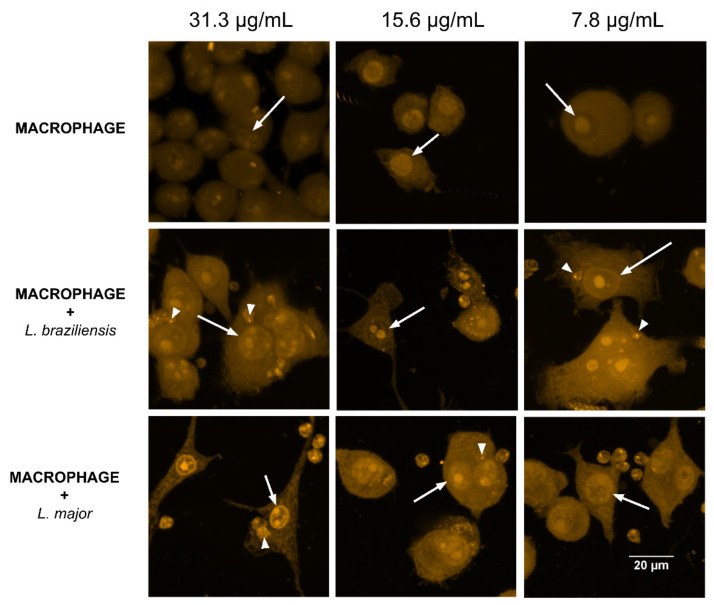
Internalization of curcumin by uninfected and infected macrophages with *L. braziliensis* and *L. major* at concentrations of 31.3, 15.6, and 7.8 µg/mL. Arrows indicate the nucleus of the macrophages, while the arrowheads indicate the amastigotes of *L. braziliensis* and *L. major* internalized in the macrophages.

The internalization of curcumin in concentrations that presented the best response in the viability test was evaluated. The analysis of the images (Figure 5) allowed us to observe that at all three concentrations curcumin was internalized in the cells since no fluorescent markers were used except for this substance. The PS distribution was observed throughout the cell cytoplasm and in the cell nucleus, suggesting that besides being dispersed in the cell, curcumin can cross the nuclear membrane (arrows). Considering that several biomolecules can be the target of interaction with the ROS produced by PDT, DNA becomes yet another cellular target for inactivation in these evaluated cells. Additionally, an accumulation of curcumin can be observed in the amastigotes internalized by macrophages infected with both species of *Leishmania* (arrowheads). This result is very important since amastigotes internalized by macrophages can also be a direct target of the ROS produced.

### 2.4. Qualitative and Quantitative Assessment of Mitochondrial Activity by Rho 123

**Figure 6:**
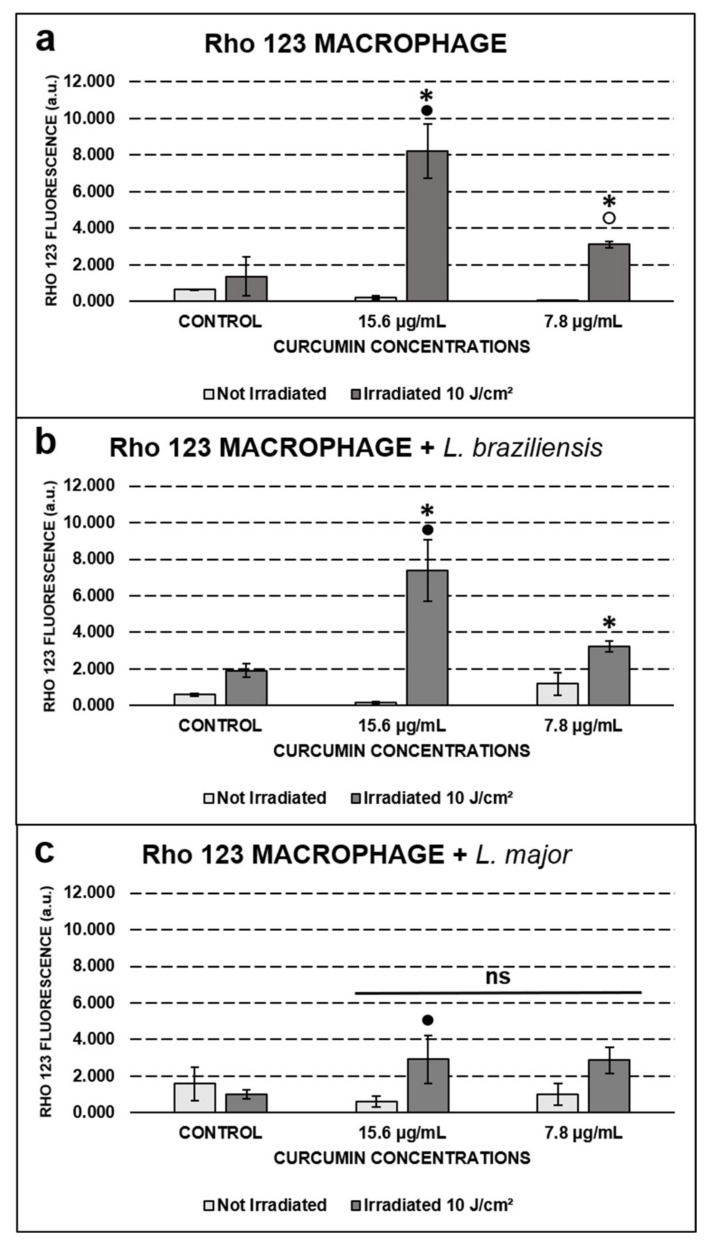
Quantification of Rho 123 fluorescence for (**a**) uninfected macrophages, (**b**) macrophages infected with *L. braziliensis*, and (**c**) macrophages infected with *L. major*. Values are expressed as mean ± SD. The * symbol represents a statistically significant difference (*p* ≤ 0.01) between the indicated groups and the non-irradiated control. The symbol “●” represents a statistically significant difference between the irradiated and non-irradiated groups at the curcumin concentration of 15.6 µg/mL. The symbol “○” represents a statistically significant difference between the irradiated and non-irradiated groups at the curcumin concentration of 7.8 µg/mL. The symbol “ns” represents the absence of a statistically significant difference (*p* > 0.05) between the control and all groups below the horizontal line.

Rho 123 is a compound that actively interacts with the mitochondrial membrane, presenting high fluorescence emissions in a broad variety of active membranes and low fluorescence in low active ones. This study showed that the controls presented less mitochondrial activity than the groups submitted to PDT (*p* ≤ 0.01), both for uninfected macrophages and for those infected with *L. braziliensis*. The fluorescence emitted by the control group was not statistically different (*p* > 0.05) from that produced by the treated groups incubated only with curcumin for the macrophages infected with *L. major*. When comparing groups submitted to PDT, mitochondrial polarization by Rho 123 was concentration dependent (*p* ≤ 0.01), showing higher fluorescence at 15.6 than at 7.8 µg/mL, for macrophages both uninfected and infected with *L. braziliensis* (Figure 6a,b).

### 2.5. Retrieval of Leishmania after PDT Exposure

**Figure 7:**
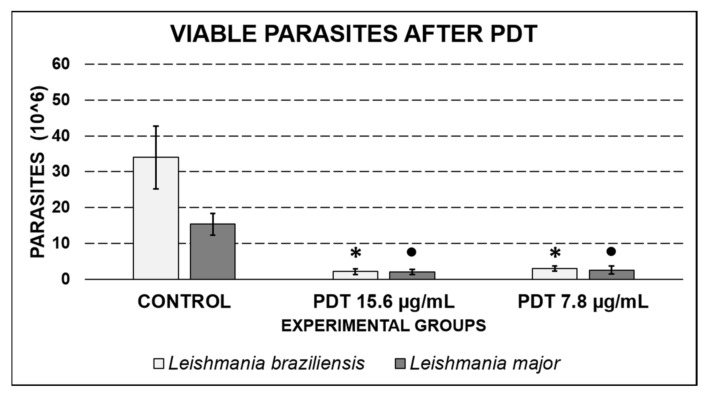
Quantitative comparison between control groups and those submitted to PDT with curcumin concentrations of 15.6 and 7.8 µg/mL for *L. braziliensis* and *L. major*. Values are expressed as mean ± SD. The symbol * represents a statistically significant difference (*p* ≤ 0.01) between the non-irradiated control group and the other groups for *L. braziliensis*. The symbol ● represents a statistically significant difference (*p* ≤ 0.01) between the non-irradiated control group and the other groups for *L. major.*

The recovery test of the post-PDT amastigote was carried out in order to verify the impact of PDT on the growth of the parasites. As can be seen in Figure 7, the control groups for *L. braziliensis* and *L. major* presented 35 × 10^6^ and 15 × 10^6^ cells/mL, respectively, while the groups submitted to PDT exhibited fewer than 5 × 10^6^ cells/mL (statistically significant decrease, *p* ≤ 0.01). There were no statistical differences in the quantification of post-PDT parasites when comparing the curcumin concentrations of 15.6 and 7.8 µg/mL (*p* > 0.05), a condition observed for both the evaluated species.

## 3. Discussion

This study showed that PDT reduced the viability of RAW 264.7 macrophages infected with *L. braziliensis* and *L. major*. In addition, we tried to interpret the mechanisms that resulted in the death of these cells, considering the potential of the mitochondrial membrane and the internalization of curcumin in the evaluated macrophages. While there is a considerable number of in vitro studies that involve the application of PDT in *Leishmania* promastigotes, especially using photosensitizers such as methylene blue and different phthalocyanines [15,16,17,18,19,20,21], the literature involving the application of PDT with curcumin is scarce, especially when it comes to assays with infected macrophages. Such a model is closer to the expected in clinical applications and in vivo, being an important contribution to the knowledge in this area.

This study showed that PDT induced a viability reduction in the groups with curcumin at 31.3, 15.6, and 7.8 µg/mL. A previous study by our research group demonstrated that macrophages infected with *Leishmania* were more sensitive to the treatment with lower concentrations of PS than parasites at the promastigote phase [22]. In order to maintain the integrity of the tissue adjacent to CL lesions, the effectiveness of the proposed protocol at low curcumin concentrations is a promising result.

In addition, cells infected with *L. braziliensis* were more sensitive to the treatment than cells infected with *L. major*, a result similar to the one observed by Oliveira and collaborators (2015), who recorded a higher efficacy of the therapy for promastigotes of *L. braziliensis* than for those of *L. amazonensis* [18]. This finding is positive for countries in South America, especially Brazil, where the *L. braziliensis* is the main species responsible for CL cases.

The SEM images showed the typical cell morphology of macrophages in the controls and in the groups treated only with curcumin, while in the groups treated with PDT, there was intense morphological change and cell destruction. Cell lysis, the loss of the rounded morphology with cytoplasmic extensions typical of macrophages, and the presence of cell fragments in the fields indicate the destruction of these cells caused by the action of PDT. Morphological changes such as the loss of the characteristic spindle-shaped morphology and the loss of cell attachments such as flagella have been described in several studies with different photosensitizers [23,24,25]. However, the literature lacks the same type of evaluation regarding infected macrophages and especially studies using curcumin. It is worth mentioning that curcumin alone was not able to cause morphological changes in the cells, at the concentrations tested, confirming its low cytotoxicity at low concentrations.

A concern arising from the results obtained in this study was to assess whether the destroyed macrophages would release viable amastigotes after the PDT treatment. The recovery test for parasites after PDT showed a lower growth rate for these parasites compared to the untreated control, indicating that the therapy was able to inactivate both the parasitized macrophages and the internalized amastigotes. Andrade and collaborators (2018) observed a 40% reduction in the number of intracellular amastigote parasites after treatment with PDT. Zinc porphyrin was used as a PS and irradiation was set at a wavelength of 455 ± 20 nm with a fluence of 90 and 180 J/cm^2^. They also demonstrated that the viability of the internalized amastigotes was compromised during the action of PDT [21].

The results obtained in the internalization of curcumin by infected macrophages reinforced the potential for photodynamic inactivation of infected macrophages and internalized amastigotes, due to the fluorescence signal of curcumin observed in intracellular parasites. Considering the small diffusion capacity and the short half-life of the ROS formed in the photochemical process of PDT, the location of the PS is closely related to its place of action [26,27,28]. In addition, the observed result reiterates what was found in our previous work [23], the binding of curcumin to the cell nucleus due to its affinity with DNA, as proposed in other studies [29,30,31].

The assessment of the mitochondrial membrane potential (Δψm) was made through the marking with Rho 123, which showed higher intensity in the groups treated with PDT. Rho 123 can be used as a marker for Δψm, indicating that in the fully active and undamaged membranes, this potential will be higher compared to the damaged ones [32,33,34].

Fedyaeva and colleagues (2014) found a correlation between the increased mitochondrial ROS production and the hyperpolarization of the mitochondrial membrane during the temperature rise [35]. ROS are produced by the mitochondrial route in order to signal and activate antioxidant cellular mechanisms. However, the high production of these species may end up resulting in a stimulation of the apoptotic cell death route [36]. Therefore, the 18-h time used in this study to evaluate the samples could perhaps coincide with this type of cellular response, not ruling out subsequent cell death from apoptosis, since most cells were not viable in the Trypan blue viability test.

The results also indicate that the macrophages infected with *L. major* did not show differences in Δψm compared to the control group. This response may be related to the different antioxidant capacities of *Leishmania* species. After all, macrophages infected with *L. major* were also less susceptible to PDT at lower concentrations of curcumin. A study performed by Tasbihi et al. demonstrated the presence of different rates of expression of mitochondrial proteins in *L. tropica* compared with *L. braziliensis*, a trait that ensures resistance to the drugs routinely used against leishmaniasis [37]. Considering this information, different species of *Leishmania* with different rates and types of antioxidant mitochondrial protein expression, such as superoxide dismutase (SOD), for example, may respond differently to PDT [38].

Although curcumin has presented toxicity to macrophages at concentrations higher than 15.6 µg/mL, the finding of greater resistance in macrophages infected with *L. major* has also been observed previously in a study by Silva and collaborators [18]. Lower viability rates were observed after the PDT treatment in macrophages infected by *L. braziliensis* compared to those infected by *L. amazonensis*. In the present study, it was observed that the changes in cell viability were confirmed by the morphological changes observed by the SEM images. The intense destruction of the cell is probably linked to the wide distribution of curcumin in its cytoplasm and nucleus of the cell, and its possible interaction with the mitochondria is revealed by the change in the patterns of Rho 123.

Since *Leishmania* is a parasite that establishes itself inside the cell and manages to evade the defense system of the host, the observation that a large number of the internalized parasites was killed by PDT after a single application is an important finding. If the therapy eliminated only the host macrophages, there would be a risk of releasing viable parasites to infect new cells. However, this study showed that alternate applications of PDT could completely eliminate the parasites from the local lesions.

## 4. Materials and Methods

### 4.1. Parasite Culture

*L. braziliensis* (M2904) and *L. major* (LV39) strains were used. Initially, the promastigote forms of the parasites were maintained in liver infusion tryptose (LIT) medium supplemented with 10% fetal bovine serum (FBS), 1% penicillin/streptomycin solution, 2 mM glutamine, 1% urine, and 2.5 mg/mL of hemine, and were peaked weekly according to the log phase of growth.

### 4.2. Cultivation of Macrophages

The macrophage strain RAW 264.7 acquired from the Rio de Janeiro cell bank was kept in 200 µL of Dulbecco’s Modified Eagle’s Medium (DMEM) supplemented with 10% FBS and 1% penicillin/streptomycin solution, and stored in an incubator at 37 °C with 5% CO_2_.

### 4.3. Macrophage Infection

For the macrophage infection, these cells interacted with *Leishmania* strains in the proportion of 10:1. First, 1 × 10^4^ macrophages were counted and transferred to each well, with a total of 24 well plates, and left for adhesion for 12 h in an incubator at 37 °C and 5% CO_2_. After that period, the parasites were counted and 1 × 10^5^ promastigotes were added to each well containing macrophages. The internalization period lasted for 24 h in an incubator at 37 °C and 5% CO_2_. Finally, the evaluation of the internalization was done by visualization in an inverted microscope Zeiss Axio Vert. A1.

### 4.4. Curcumin Preparation

Curcumin (PDT Pharma) stock solution was initially diluted in dimethylsulfoxide (DMSO) at the concentration of 100 mg/mL. Subsequently, a 1:10 dilution in alcohol was made to obtain a 10 mg/mL curcumin solution in DMSO/alcohol. The work solutions for the experiments were prepared by diluting the stock solution in phosphate-buffered saline (PBS), and the highest concentration was diluted in a 1:20 ratio. The final proportion of DMSO in the working solutions was lower than 1%.

### 4.5. Photodynamic Therapy

All the tests were performed in triplicate, keeping the experimental groups sheltered from light. The experimental groups were divided into non-irradiated (control and curcumin groups without irradiation at different concentrations) and irradiated (irradiated control group and PDT groups with curcumin at different concentrations). After preparing the infected culture described in topics *4.2* and *4.3*, 200 µL of working solutions of curcumin were added directly into culture wells without medium, remaining in incubation (37 °C and 5% CO_2_) for 1 h. The control and irradiated control groups were incubated with PBS under the same conditions. An LED-based device (Biotable Irrad/LED) was used for irradiation, with a wavelength of 450 ± 5 nm, power of 3 W per LED, irradiance of 36 mW/cm^2^, and fluence of 10 J/cm^2^. The irradiation time was set up in 278 s. The viability and mitochondrial potential tests were carried out 18 h after the irradiation of the experimental groups. Until then, the cells were incubated with 200 µL DMEM in an incubator at 37 °C and 5% CO_2_.

### 4.6. Viability Assessed by the Trypan Blue Exclusion Test

The Trypan blue exclusion test is based on the property of living cells to eliminate the dye, while dead cells lose the selective ability of their membranes and tend to accumulate the compound in the intracellular medium. The test was performed 18 h after the PDT application, adding a Trypan blue solution (0.2%) followed by a five-minute incubation period. Viability was assessed by counting the number of living and dead cells in 10 different random fields for each well.

### 4.7. Morphological Evaluation by Scanning Electron Microscopy

The three lowest curcumin concentrations evaluated in the viability test were selected to assess the morphology of the macrophages after PDT using SEM. The cells observed under SEM were initially fixed with 2.5% glutaraldehyde solution diluted in PBS with 0.9% saline and pH 7.4. After fixation and post-fixation with osmium tetroxide solution, dehydration was carried out, immersing the coverslips for 10 min successively in baths with 30, 40, 50, 60, 70, 80, 90, and 100% ethanol, the latter being repeated twice. Finally, a drop of hexamethyldisilazane (HMDS) was added, followed by drying in an incubator.

The samples were metalized with gold and analyzed using a scanning electron microscope Zeiss EVO MA 10.

### 4.8. Curcumin Internalization in Macrophages Isolated and Infected with Leishmania

The process of curcumin internalization started with the culture and infection of macrophages performed in glass coverslips arranged in 24 well plates. The internalization of curcumin at 31.3, 15.6, and 7.8 µg/mL was evaluated. The incubation time was one hour, followed by washing with PBS and fixing with 4% paraformaldehyde for 15 min at room temperature. After fixing, the coverslips were again washed with PBS and mounted on glass slides. The analysis was performed under a Zeiss LSM 700 confocal microscope.

### 4.9. Evaluation of Mitochondrial Activity by Rhodamine 123 Fluorescent Staining

Rho 123 is a cationic fluorescent green dye that tends to accumulate in the mitochondria without having a cytotoxic effect, being used to assess the potential of the mitochondrial membrane in apoptotic cells. For this evaluation, macrophages were grown in circular coverslips and subsequently infected with *Leishmania* species evaluated in this study. After a period of incubation of 18 h after PDT, the groups were labeled with 20.0 µg/mL of Rho 123 for 40 min at room temperature. Subsequently, the slides were prepared using Prolong Gold Antifade with DAPI (Thermo Fisher) and evaluated using a Zeiss LSM 700 confocal microscope. The quantification of the emitted fluorescence was performed in triplicate using the ImageJ software, maintaining the microscope acquisition parameters for all the images obtained in the experiment.

### 4.10. Retrieval of Leishmania after PDT

In order to evaluate the proliferative capacity of the remaining parasites in the culture after PDT application, the parasites found in the groups treated with PDT (curcumin at 15.6 and 7.8 µg/mL) were retrieved, quantified, and compared to the control group. In order to do so, macrophages were lysed with a 0.05% SDS solution dissolved in supplemented LIT medium and subsequently incubated in an incubator at 27 °C for 48 h. After the incubation period, the promastigotes obtained were counted in a Neubauer chamber.

### 4.11. Statistical Analysis

The ANOVA test was applied using the BioEstat 5.0 software from Mamirauá Institute (Tefé-AM, Brazil), used for all the quantitative analysis of this study. All tests were performed in three independent triplicates.

## 5. Conclusions

Therefore, curcumin showed a good distribution inside the macrophages, as well as in the amastigotes within them, at the tested concentrations, after an hour of incubation. PDT with curcumin was able to reduce the viability of amastigotes within the macrophages, besides changing their mitochondrial activity, which resulted in a significant impact on the morphology of these cells, causing intense destruction. Although this study is an in vitro evaluation of PDT with curcumin and in vivo studies are required to improve this research field, an interesting point is the low recovery of parasites after the PDT treatment, which allowed us to infer that the use of PDT associated with curcumin, at the tested parameters, was effective in eliminating the parasites and reducing the parasitic load with a single application. Protocol adaptations for in vivo application, serial applications of PDT, and development of specific pharmaceutical formulations for topical applications of this treatment, may be future fields of study to advance this research.

## Data Availability

The data presented in this study are available on request from the corresponding author.
